# Automated Prostate Cancer Detection on T2-Weighted MRI Using a Dual-Stream Attention Network: A Study on Private Saudi Clinical Data and Public Benchmark Datasets

**DOI:** 10.3390/jcm15093327

**Published:** 2026-04-27

**Authors:** Saeed Alqahtani, M. A. Jowhari, Yahya.Q. Sabi, Hussein Alshaari

**Affiliations:** 1 Radiological Sciences Department, College of Applied Medical Sciences, Najran University, Najran 61441, Saudi Arabia; 2 Health Research Center, Najran University, Najran 61441, Saudi Arabia; 3 Radiology Department, Prince Mohammed bin Nasser Hospital (PMNH), Ministry of Health (MOH), Jazan 82911, Saudi Arabia

**Keywords:** prostate cancer, deep learning, dual-stream network, attention mechanism, medical image classification, magnetic resonance imaging (MRI)

## Abstract

**Background:** The steady rise of prostate cancer in Saudi Arabia signals a critical public health shift that requires immediate investment in early detection and prevention to mitigate a future clinical crisis. Accurate diagnosis using multiparametric MRI and PI-RADS scoring remains challenging, as interpretations are highly experience-dependent and subspecialized radiologists are limited. **Methods:** To address this gap, this study introduces a novel Dual-Stream Attention Network designed to automate the classification of low-risk (PIRADS 2-3) versus high-risk (PIRADS 4-5) lesions from T2-weighted MRI. Leveraging a ResNet50 backbone, the architecture employs parallel streams for Local and Global Feature Processing, each enhanced by a Channel-Spatial Attention module to highlight diagnostically relevant regions. These features are integrated through a Cross-Stream Fusion mechanism and a gate-controlled Adaptive Feature Fusion module to optimize multi-scale information. The model was developed and validated on a regional dataset of 3850 images from Jazan Specialist Hospital and Prince Mohammed bin Naser Hospital. This research provides a standardized, high-precision diagnostic path tailored to the Saudi Arabian population, conducted under institutional review board approval (No. 25138). **Results:** The proposed dual-stream attention network achieved an accuracy of 97.8% on the validation set and 96.4% on the test set, demonstrating high performance and generalization capabilities in classifying prostate lesions from Saudi patient populations. **Conclusions:** The proposed dual-stream architecture with novel attention and fusion mechanisms demonstrates high effectiveness for prostate cancer classification from T2-weighted MRI in Saudi clinical settings. This represents the first deep learning model specifically trained and validated on Saudi Arabian prostate MRI data, with the potential to address the shortage of specialized expertise and improve diagnostic efficiency in the Kingdom.

## 1. Introduction

Prostate cancer (PCa) is a common urological malignancy in middle-aged to older men worldwide, causing 375,304 deaths and 1.4 million new cases in 2020 [1,2]. The prostate gland, located below the bladder and near the rectum, produces fluid that combines with sperm to form semen. PCa develops through abnormal cell growth in the prostate gland and can invade surrounding tissues. Adenocarcinomas are the most common type, with other variants including sarcomas, transitional cell carcinomas, squamous cell carcinomas, neuroendocrine tumors, and small cell carcinomas [3].

Common PCa symptoms include urinary dysfunction, bone pain, fatigue, hematuria, hematospermia, numbness, jaundice, weight loss, and erectile dysfunction [4]. Risk factors include age, family history, obesity, ethnicity (particularly African men), and genetic predisposition [5]. While PCa often grows slowly, aggressive cases can metastasize to surrounding tissues. Therefore, early and accurate diagnosis is critical for timely intervention and treatment.

Traditional PCa detection relied on PSA testing and digital rectal examination, but these manual methods are time-consuming and error-prone [6,7]. Multiparametric MRI (mpMRI) replaced TRUS as the first-line imaging modality, offering superior soft-tissue resolution and comprehensive prostate analysis [8,9]. The prostate imaging-reporting and data system (PI-RADS) standardizes mpMRI interpretation [10]. Combined, mpMRI and PI-RADS reduce overdiagnosis, improve staging accuracy, and guide biopsies [11].

While mpMRI is efficient for PCa diagnosis, manual interpretation is challenging and time-consuming. Computer-aided diagnosis systems and machine learning (ML) algorithms address these limitations for improved diagnosis and treatment. ML techniques extract lesion features from imaging modalities, including ultrasound, MRI, and CT scans [12]. Features such as size, shape, intensity, and texture are crucial for distinguishing malignant from benign tissues [13]. Popular ML methods for PCa identification include KNN, fuzzy logic, random forest, decision trees, SVM, ANN, Naïve Bayes, genetic algorithms, and LSSVM [14,15,16,17]. These techniques perform well on small to medium-sized datasets but struggle with complex or noisy data and generalization challenges [18].

Deep learning (DL) addresses traditional ML generalization problems by extracting complex features from medical imaging data. Convolutional Neural Networks (CNNs) demonstrate promising performance in healthcare imaging applications [19]. Recent approaches include CNN-based models like C-Net using cascaded networks [20]. To enhance model efficacy, researchers employ transfer learning, loss function refinement, attention mechanisms, and diverse CNN architectures [21,22]. Pre-trained configurations such as VGG-16, ResNet, ShuffleNet, SqueezeNet, ImageNet, DenseNet, VGG-19, GoogleNet, Inception-v3, and EfficientNet are applied to large medical imaging datasets [23].

Pre-trained CNN architectures reduce model construction time and computational resources for detection, classification, and segmentation tasks. Beyond pre-training, Knowledge Distillation has emerged as a vital technique for transferring complex feature representations into simplified models, facilitating model compression and enhancing performance in scenarios with limited or weakly supervised medical data [24]. Recent studies employ ResNet, dual optimizers (Adam and SGD), and Faster R-CNN for efficient PCa detection using MRI data [25]. Dual optimizers improve performance through fixed learning rates during training. A CNN-based fusion model incorporating CPCB and SE-Net modules achieved 90.7–94.4% accuracy for Gleason-score grouping in PCa staging [26]. Additionally, CNN-based AI classifies prostate lesions with performance comparable to expert radiologists [27], while U-Net deep learning achieves diagnostic performance equivalent to PI-RADS assessment for detecting clinically significant prostate cancer [28].

A hybrid LeNet-MobileNetV2 and VGG-16 CNN model achieved 96.3% accuracy for PCa detection, reducing complexity and overcoming generalization problems [29]. A 3D dense multipath CNN on segmented MRI images achieved Dice similarity coefficients of 95% and 89% on independent test datasets [30]. However, CNNs act as black boxes, limiting clinical interpretability and trust. They also struggle with overfitting, noisy data, and distinguishing subtle Gleason patterns in heterogeneous tissue [31]. Furthermore, addressing data heterogeneity remains a significant hurdle in medical image analysis; recent advancements in Federated Learning, such as perceptual hashing hypernetworks, have been proposed to better balance model personalization with global generalization across diverse clinical sites [32]. To address these limitations, advanced methods including Vision Transformers (ViTs), Auto-encoders, Ensemble models, and Generative Adversarial Networks (GANs) have been introduced to medical imaging [33,34].

ProstateNET, a multi-encoder-cross-attention-fusion framework, achieved 91% AUC for PCa identification and classification [35]. A Vision Transformer (ViT)-based network capturing local and global contextual features achieved a 96.7% kappa score for prostate lesion detection [36]. A hybrid CNN+ViT approach addressed data imbalance through fine-tuning, data augmentation, and dual synthetic oversampling (SMOTE and ADASYN), achieving 95.32% and 97.6% Matthews Correlation Coefficient, respectively [37]. Preprocessing techniques, including contrast enhancement, edge preservation, and noise reduction, combined with GLCM feature extraction, improved performance.

In this work, we investigate automated prostate cancer classification using T2-weighted (T2W) MRI sequences. Although multi-parametric protocols often include Diffusion-Weighted Imaging (DWI) and Apparent Diffusion Coefficient (ADC) maps, we restricted our analysis to T2W images to evaluate the model’s performance on the most common anatomical sequence. To validate this approach, the proposed framework was tested on three datasets: an institutional cohort (n = 75), the PI-CAI dataset, and the Prostate158 dataset. For all three datasets, only the T2W sequences were utilized in the training and testing phases. This experimental design ensures that the results are comparable across the different cohorts regardless of the availability of functional imaging modalities.

This research makes the following contributions to the field of automated prostate cancer detection:

- Developed a novel dual-stream deep learning framework incorporating Channel-Spatial Attention and Adaptive Feature Fusion. This architecture enables the autonomous extraction of multi-scale features, eliminating the need for traditional, labor-intensive handcrafted feature engineering.
- Improved diagnostic sensitivity for early-stage prostate cancer by implementing simultaneous multi-scale feature processing. This approach successfully identifies subtle, fine-grained pathological patterns that are frequently missed by conventional single-scale models.
- Validated model performance using a localized dataset of Saudi Arabian patients, achieving a high test accuracy of 96.4% and an AUC of 0.99. These results demonstrate the model’s robustness and its high potential as a reliable clinical decision support tool.

The remainder of this paper is organized as follows: Section 2 details the dataset sources, configuration, and ethical board approval. Section 3 outlines the proposed model’s classification pipeline. Section 4 presents the experimental results and discussion, and Section 5 concludes the paper.

## 2. Data Source and Ethical Approval

The dataset used in the current study consists of private medical imaging data obtained in Jazan Specialist Hospital and Prince Mohd bin Naser Hospital. The ethical approval was granted through institutional approval No. 25138, dated 9 October 2025.

All patient information was covered with anonymization before analysis, and the identities of each patient were substituted with the sequence of patient codes (PAT_0000, PAT_0001, etc.), so that the information was fully de-identified and patient privacy was not compromised. The collection and use of the datasets were in strict compliance with the guidelines of the Health Insurance Portability and Accountability Act (HIPAA) and the requirements of the institutional review board of the locality.

All regional data were acquired using a standardized clinical setup on a Siemens MAGNETOM Aera system (1.5 Tesla, serial number 42552, Siemens Healthineers, Erlangen, Germany). This is a 1.5 Tesla scanner equipped with Tim (Total Imaging Matrix) technology and the DOT (Day Optimizing Throughput) system.

### 2.1. Dataset Composition and Structure

The study utilized a comprehensive dataset comprising 130,343 medical images. To maintain clinical relevance, the data were stratified by PI-RADS risk groups, as shown in Table 1.

Analysis of the cohort revealed that across 75 patients scanned between 2022 and 2025, a total of 1183 MRI series and 130,343 slices were acquired. Annual cohort sizes were balanced, ranging from 7 to 12 patients per year. The average number of slices per series (~110) reflects standard acquisition practice, where slice count is determined by the extent of the prostate gland.

The hierarchical structure maintains valuable metadata regarding patient categorization, time frame of collection, study sessions, imaging series, and individual image objects. The data includes various suspect cohorts collected at varying time periods, providing temporal diversity and minimizing possible batching effects.

#### 2.1.1. Patient Cohort and Clinical Categories

The dataset contains multi-parametric MRI images of patients classified under the PI-RADS scoring guidelines. PI-RADS is a standard parametric scoring system utilized to evaluate the risk of clinically significant prostate cancer on the multi-parametric MRI images, with a score ranging from 1 to 5. In this binary classification study, the patients had been categorized into two groups:

- **Low Suspicion (PI-RADS 2-3)**: Patients with low to intermediate suspicion of clinically significant prostate cancer (Label: 0).
- **High Suspicion (PI-RADS 4-5)**: Patients with a high suspicion of clinically significant prostate cancer (Label: 1).

#### 2.1.2. MRI Imaging Protocol

Multi-parametric MRI tests were performed using clinical-grade MRI scanners and in accordance with the standardized imaging procedures. All the patients underwent multi-parametric prostate MRI, which usually consists of several imaging sequences, including T2-weighted imaging, diffusion-weighted imaging (DWI), dynamic contrast-enhanced (DCE) imaging, and an apparent diffusion coefficient (ADC) map. For this research study, we particularly focused on T2-weighted imaging sequences, which are very beneficial in terms of soft tissue contrast and are essential for anatomical delineation of the prostate gland and the identification of possible lesions. T2-weighted sequences are essential to PI-RADS scoring and provide better visualization of zonal anatomy and pathological processes.

#### 2.1.3. Image Selection and Filtering

Out of the detailed multi-parametric MRI data of 130,343 total image slices of all the sequences and orientations, we employed a systematic filtering process in order to select clinically relevant T2-weighted imaging data. They were selected according to the description of the DICOM series that indicates certain T2-weighted acquisition protocols.

The T2-weighted series provided in the research included a variety of acquisition parameters and orientations, all of them being captured in the transverse (axial) plane, the typical orientation of prostate imaging. Table 2 presents the distribution of the selected T2-weighted series.

The filtered dataset of 3850 T2-weighted images represents clinically standardized acquisitions that could be used in automated analysis but with the diversity of protocols, so that the model could be more generalized. Several T2-weighted protocols were included (fat suppressed, turbo spin echo, HASTE) in order to ensure that the model learns strong features that are not dependent on certain acquisition parameters.

The final selected dataset utilized for the model development includes:

- **Total images**: 3850 T2-weighted MRI slices
- **Patient cases**: 75 unique patients (36 PI-RADS 2-3, 39 PI-RADS 4-5)
- **Collection period**: 2022–2025
- **Imaging modality**: Multi-parametric MRI (T2-weighted sequences)
- **Class distribution**: Approximately balanced (48% vs. 52%)
- **Clinical annotation**: Expert-validated PI-RADS scoring

The dataset represents a solid foundation for DL model development and validation to predict the automated multi-parametric MRI-based prostate cancer risk stratification.

## 3. Proposed Architecture: Dual-Stream Attention Network

### 3.1. Architecture Overview

**Figure 1:**
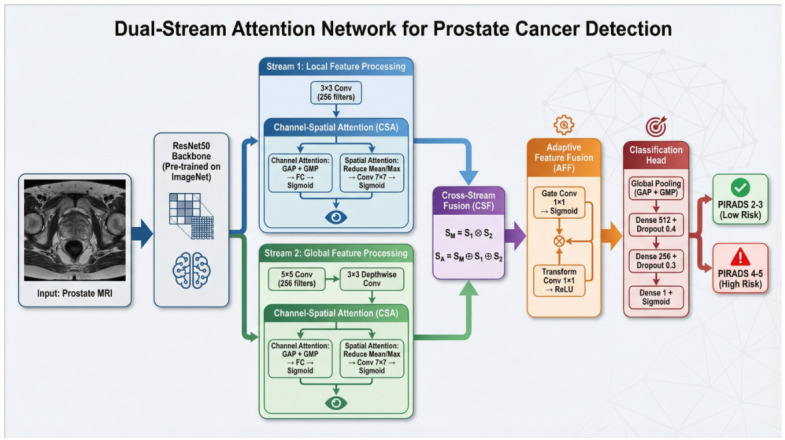
Architecture of the Dual-Stream Attention Network for Prostate Cancer Classification.

The proposed Dual Stream Prostate Cancer Detection (DSPCD) model comprises two novel modules: Channel-Spatial Attention (CSA) and Adaptive Feature Fusion (AFF). The architecture (Figure 1) begins with a ResNet50 backbone pre-trained on ImageNet, followed by two parallel processing streams. Stream 1 (S1) uses a standard convolution and CSA, while Stream 2 (S2) employs a 5 × 5 convolution followed by depthwise convolution and CSA. The CSA module integrates both spatial attention (using reduce mean and reduce max operations followed by concatenation and convolution) and channel attention (using global average pooling and global max pooling with fully connected layers). Cross-Stream Fusion (CSF) combines the two streams through element-wise multiplication and addition. The Adaptive Feature Fusion module employs learnable gating through parallel gate convolution (sigmoid) and transform convolution (ReLU) paths, followed by element-wise multiplication. The classification head consists of global pooling (GAP + GMP concatenation), dense layers with dropout regularization, and sigmoid activation for binary classification of prostate MRI into PI-RADS 2-3 (low risk) or PI-RADS 4-5 (high risk) categories.

### 3.2. Backbone Feature Extractor

The network uses the ResNet50 model, which is pre-trained on ImageNet as the backbone feature extractor. This backbone is used as the basis of deriving hierarchical feature representations of input medical images. The pre-trained weights offer strong initial features, which are further refined to the medical imaging field with transfer learning approaches.

### 3.3. Dual-Stream Processing Architecture

The fundamental innovation of our architecture is the dual stream design, which processes features on more than one scale, just like the diagnostic process of medical professionals as they scan small parts of the image and the whole image at the same time.

#### 3.3.1. Stream 1: Local Feature Processing

The first stream focuses on fine-grained local features that are useful in the detection of the subtle pathological patterns. This stream uses fewer receptive fields to target local image areas and does not lose spatial details that can be of diagnostic interest. The features obtained using this stream are highly responsive to texture changes, boundary definitions, and minor abnormalities that may be manifestations of a pathological state.

#### 3.3.2. Stream 2: Global Feature Processing

The second stream is designed to include wider contextual data and interpret the overall spatial arrangement of the anatomical structures. With the use of larger receptive fields, this stream gives a holistic view of the image through the identification of global patterns and relations between various regions. This stream is imperative to the general context, where the local features can be seen and will help to minimize any false positives by looking at the larger anatomical context.

The concurrent processing of both streams allows the model to retain a comprehensive understanding of the medical image at different scales, having detailed local analysis interactions and a global contextual view.

### 3.4. Novel Attention Mechanisms

**Channel-Spatial Attention Module:** We propose a Channel-Spatial Attention module, which allows the model to pay more attention to the features that are diagnostically relevant and reduce noise and non-relevant information. This module functions with the help of two inter-operative attention mechanisms.

The feature channels with the most diagnostically valuable information are obtained by comparing the mean and maximum activation values across spatial dimensions using the attention module. The mutually exclusive feature importance is captured using this dual-statistics approach; average pooling identifies features with uniformly activated patterns, and max pooling highlights the features with salient peaks. The channel importance signal is then coded in a smaller dimensionality representation before restoring the full channel attention map, thus allowing the efficient computation whilst maintaining discriminative effectiveness.

At the same time, the spatial-attention branch identifies those spatial locations in the image that should receive more focus. The module combines information on all channels at every spatial location to create an attention map, which identifies regions of interest. The spatial attention, by the average and maximum channel pooling, attains the unique types of spatial salience. The final output is the product of the multiplicative process of both attention mechanisms to the input features, which essentially weights the single feature elements in proportion to their importance in the channel and spatial dimensions. This output is formalized as follows in Equation (1).

(1) $$F_{out}=F_{in}⊙A_{channel}⊙A_{spatial}$$

where $F_{in}$ represents the input features, $A_{channel}$ denotes the channel attention map, $A_{spatial}$ represents the spatial attention map, and ⊙ indicates element-wise multiplication. This interaction guarantees that this model puts an emphasis on those features that are both semantically significant (channel attention) and spatially significant (spatial attention). The output representation will aid in making more discriminative characterizations of the medical image classification task.

**Adaptive Feature Fusion Module:**

The Adaptive Feature Fusion Module is aimed at addressing the challenge of incorporating representations obtained by different stages of processing or spatial resolutions. Unlike primitive concatenation or addition functions, this module is an adaptive learner that dynamically adjusts the weighting of the contribution of each source of features with respect to its contribution to the classification task.

The module applies a gating mechanism that estimates the significance of different components of features dynamically. A special gating network is actually trained to produce weighting factors of every feature element, and a parallel transformation network is used to recast the input features to a shared representation space. As a form of adaptive fusion, the formulated expression is described in Equation (2).

(2) $$F_{fused}=T\left(F\right)⊙G\left(F\right)$$

where T(·) represents the transformation function, G(·) denotes the gating function, and F is the input feature map.

The gate values provide the capability of the flow of the information as soft switches that enable the model to emphasize the feature components that are relevant to the present input and reduce those that provide less discriminative capacity.

Such adaptive approach is particularly useful in the field of medical imaging, where the diagnostic relevance of various elements could differ greatly with respect to the specific pathology being examined. The model can achieve better robustness and accuracy in its classification outputs by training to modify its gating approach based on characteristics of the input.

### 3.5. Cross-Stream Fusion Strategy

To be able to take the complementary benefits of the dual processing streams, the outputs of the streams should be combined productively. Our cross-stream fusion approach, utilized in this case, is better than mere concatenation as it fully describes the interaction between the two streams.

The fusion process is implemented in three stages in a series. The two streams are first multiplied on an element-wise basis thus encoding the interactions that exist between them and identifying the features that are mutually reinforced at both local and global scales. The entire fusion can be formulated as Equation (3).

(3) $$F_{fused}=S_{1}+S_{2}+\left(S_{1}⊙S_{2}\right)$$

where $S_{1}$ and $S_{2}$ represent the features from Stream 1 and Stream 2, respectively. Second, the three constituent elements, features of Stream 1, features of Stream 2, and their interaction term are integrated additively, allowing no information to be lost, and at the same time, they capture the synergistic effects. Third, the fused representation is normalized to stabilize its feature distribution before it is passed to the Adaptive Feature Fusion module, which further refines the combined features.

Multi-stage fusion approach will ensure that the final representation reflects the individual capabilities of each stream as well as the synergy of the two streams and thus provide a set of features that bring about a complete set of features that are amenable to correct classification.

### 3.6. Classification Head

The rich feature representation obtained in the dual-stream network is converted into an ultimate diagnostic decision by the classification module. To derive different global statistics out of the spatial feature maps, the classification head uses two complementary global pooling strategies that include average pooling and max pooling. Average pooling represents the sum total of the activation level throughout the image, and max pooling represents the most dominant responses, which reflect the most salient pathological signals. The dual pooling functioning can be stated as Equation (4).

(4) $$v_{global}=\left[\mathrm{GAP}\left(F_{fused}\right)\cdot \mathrm{GMP}\left(F_{fused}\right)\right]$$

Here, GAP and GMP refer to Global Average Pooling and Global Max Pooling, respectively, and [·] is concatenation. The pooled features are joined together to create a holistic global descriptor. This descriptor is then further passed onto several fully connected layers of progressively decreasing dimensionality. This kind of hierarchical processing makes it possible for the model to learn more abstract and task-specific representations.

To reduce overfitting, dropout regularization is used, which involves randomly turning off neurons during training to make the network learn strong features that generalize well to unknown data. The final layer produces a probability score indicating the likelihood of the positive class as calculated in Equation (5).

(5) $$p=\sigma (W^{T}h+b)$$

With σ(·) the sigmoid activation function, h the activations of the last hidden layer and W and *b* the learned weights and bias, respectively.

### 3.7. Model Summary

The Dual-Stream Attention Network is a combination of a set of architectural elements specific to medical image classification. The total number of parameters is 41,976,967 (160.13 MB), which includes 41,923,335 trainable ones (159.92 MB) as summarized in the Table 3.

## 4. Results

### 4.1. Dataset and Training Configuration

The data was divided into 69.82% training (2688 images, 42 batches), 14.96% validation (576 images, 9 batches), and 15.22% test (586 images, 10 batches) sets, where the batch size was 64. This stratified division was applied to prevent leakage of data as well as enable strict evaluation of the model. The binary classification approach differentiated between low-risk (PIRADS 2-3) and high-risk (PIRADS 4-5) cases, 275 and 311 samples of the test set, respectively, and 287 and 289 samples of the validation set, respectively. The near-equal distribution (53.1% high-risk in the test set and 50.2% high-risk in the validation set) is beneficial; balanced classes guarantee that the performance metrics would be a representation of actual model performance and not a predilection to a particular category.

Model training was based on two-stage transfer learning. In Stage 1 (epochs 1–25), the ResNet50 backbone was frozen to retain ImageNet-learned features, and only the dual-stream attention modules and classification head were optimized using the Adam optimizer, with a learning rate of 0.001 and binary cross-entropy loss. The validation area under the curve (AUC) was monitored during early stopping using a 7-epoch patience, and the learning rate was halved after three consecutive plateaued epochs (factor = 0.5). In stage 2 (epochs 26–50), the layers of ResNet50 were unfrozen for fine-tuning, and the learning rate was set to 0.0001, a solid 10 times less than the original, which reduced perturbation of pre-trained weights. The early-stopping patience was increased to 10 epochs, and the learning rate reduction patience was increased to 7 epochs at this stage, as it was feasible to allow the model more flexibility to fine-tune task-specific representations without necessarily overfitting to the relatively small medical data.

### 4.2. Training Dynamics and Convergence

**Figure 2:**
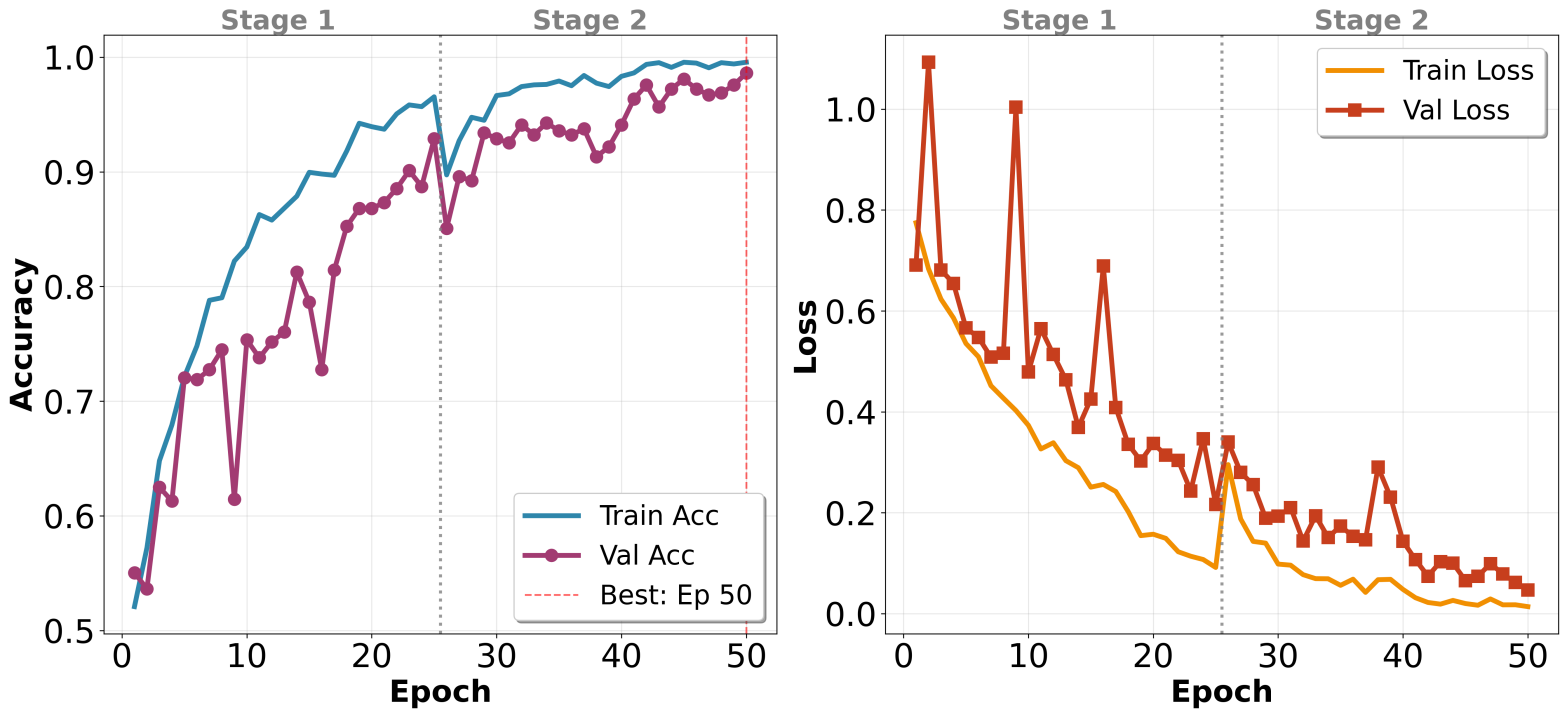
Training and Validation performance for 50 epochs as shown by the accuracy (**left**) and loss (**right**) curves. The vertical dashed line at epoch 25 indicates the transition from Stage 1 to Stage 2. The model backbone is ’frozen’ to stabilize initial learning. In Stage 1, the backbone of the model was frozen, and in Stage 2, the entire model was fine-tuned for better performance.

The model was shown to have progressive learning in both training phases, as illustrated in Figure 2. Stage 1 witnessed the training accuracy increasing from around 50% at epoch 25 up to 94%, and validation accuracy increasing more slowly, between 52% and 91%. The difference between training and validation metrics suggests that it is over-fitting, even though the overall training performance is high. The training and validation losses reduced gradually, between 0.8 and 0.15 and 0.75 to 0.20, respectively, which showed that the frozen-backbone strategy was successful in extracting discriminative features of the convolutional layers.

Stage 2 stabilized this trend and further refined the unfrozen layers. The training accuracy rose progressively from 90% to 99%, and the validation accuracy rose progressively from 88% to 98%, with losses stabilizing near the Stage 1 endpoints. The epoch 25 transition, which is indicated by the vertical dashed line in the curves, proved that the lower learning rate and the selective layer unfreezing helped avoid the volatile behavior that occurs when fine-tuning is initiated. There were no cases of sudden loss spikes and lowered accuracy, which means that Stage 1 created a strong base of adaptation. This convergence trend confirms the two-stage procedure as a balanced approach to strengthen learning and reduce overfitting over the entire 50-epoch training process.

### 4.3. Overall Classification Performance

The model produced strong results in both sets, with an accuracy of around 96.0% on the test set (586 correctly classified out of 565 observations) and around 98.0% on the validation set (563 correct classifications out of 576 observations). The 2% difference in performance is within the normal range of expectations and can be related to the common phenomenon according to which validation accuracy is slightly higher than the test accuracy, as optimization gains are achieved in subsequent early stopping and learning-rate adjustments. The small margin between the results shows that the model has learned general features rather than just memorizing (overfitting) patterns specific to this dataset. If the model had overfitted, we would have seen much larger differences or inconsistent performance across the groups.

As seen in Table 4, there is an exceptionally well-balanced performance in risk categories. The low-risk (PIRADS 2-3) and high-risk (PIRADS 4-5) classes achieved exactly the same values on the test set: 0.96 precision, 0.96 recall, and 0.96 F1-score. This symmetry is important in practice, as it indicates that the model does not favor one category of observation, even though the high-risk category makes up 53.1% of the total test observations and could otherwise bias predictions towards the majority categories. Even higher results were obtained in the validation set, with low-risk precision of 0.99, recall of 0.98 and an F1-score of 0.98, as well as high-risk precision of 0.98, recall of 0.99 and an F1-score of 0.98. Both sets gave the same macro and weighted-F1 scores (0.96 in test and 0.98 in validation), indicating that improvement in performance is due to enhancement in both risk categories and not due to one class outperforming the others.

### 4.4. Confusion Matrix Analysis and Diagnostic Performance Indicators

**Figure 3:**
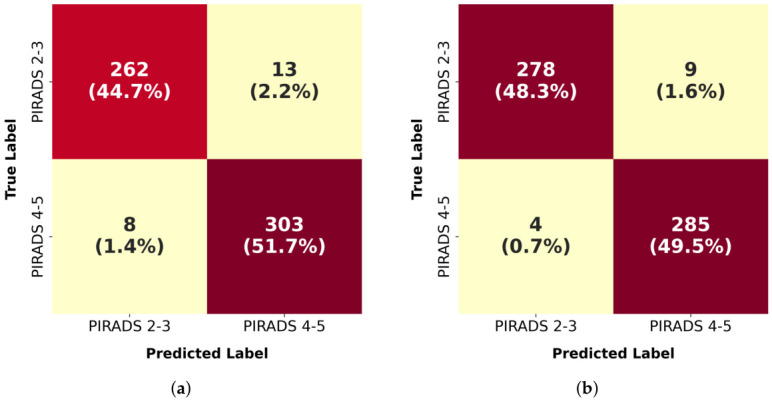
Confusion matrices showing the classification results in (**a**) the test dataset and (**b**) validation dataset.

The confusion matrices shown in Figure 3 provide an understanding of the prediction pattern of the model, as well as the derivation of clinically relevant diagnostic measures. The model accurately identified 262 cases of low-risk (true negatives) and 303 cases of high-risk (true positives) in the test set with a combined accuracy of 96.4%. The confusion matrix also showed 13 false positives (2.2%) where PIRADS 2-3 were flagged as PIRADS 4-5 and 8 false negatives (1.4%) where PIRADS 4-5 were missed. These values correspond to a sensitivity of 97.4% in identifying high-risk cases and a specificity of 95.3% in detecting low-risk cases.

The validation set was even more successful with 278 cases and 285 cases being correctly classified as low risk and high risk, respectively, with only 9 false positives and 4 false negatives. This gave a sensitivity of 98.6% and a specificity of 96.9%, which reported an improvement of around 1.2% and 1.6% over the test set, respectively. The false positive and false negative rates of the validation set were lower than those in the test. The uniform high consistency of all the diagnostic indicators aligns with the overall accuracy trends and probably shows minor distributional differences between the sets as opposed to systematic model bias.

### 4.5. ROC Curve Analysis

**Figure 4:**
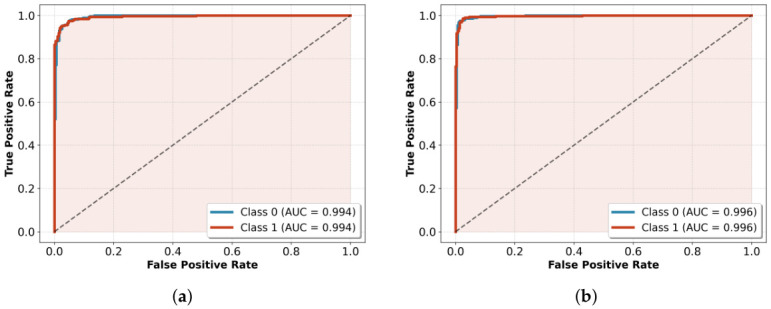
ROC curves for (**a**) the test dataset and (**b**) validation dataset, which show an excellent discriminative power with an AUC value of 0.994 and 0.996, respectively.

ROC curves in Figure 4 evaluate the discriminative ability of the model on the complete range of decision thresholds, and not at a fixed 0.5 cutoff. The ROC area under the curve (AUC) is a summary of general performance on a scale of 0.5 (random chance) to 1.0 (perfect discrimination). The model achieved a high AUC of 0.994 for both low- and high-risk groups. This means that 99.4% of the time, the model correctly ranked a high-risk case above a low-risk one.

There was a minor improvement in the validation set with an AUC of 0.996 across both classes, a difference of only 0.002 (with the test set). This strong correlation of the training and validation sets, as well as the test set performance, confirms that the discriminative capacity of the model generalizes consistently to data not used in training, whereas a minor difference in performance across sets indicates consistent performance without preference towards any risk type.

### 4.6. Comparison of Test and Validation Performance

The model showed stability in both sets and that the changes are not significantly great, indicating and suggesting a strong generalization. Validation accuracy was marginally higher than test accuracy (98.0% and 96.0%, respectively), which is to be expected and acceptable considering that validation data is used to make early stopping and learning-rate adjustments in training. Of more significance, threshold independent measures showed impressive stability: the AUC curve was differentiated by a range of 0.002 (0.996 on validation versus 0.994 on test), and average precision was differentiated by a range of 0.002–0.003 (0.996–0.997 on validation versus 0.993–0.995 on test). These insignificant discrepancies indicate that the nature of the model in terms of its discriminative ability and precision recall trade-off is not dependent on the dataset, but it is indicative of over-fitting artifacts.

The error rates and diagnostic measures had a similar tight correlation. The false-negative rate (1.4%) and false-positive rate (3.1%) of the validation set specifically, were slightly better than the test rates (2.6% and 4.7%, respectively), but the sensitivity (98.6% versus 97.4%) and specificity (96.9% versus 95.3%) were marginally higher, but the difference, probably due to natural distributional variation between sets, is only 1.2–1.6 percent points. Notably, none of the test-set measures achieved a lower accuracy or F1-score (0.96), which gives a high performance floor.

### 4.7. Five-Fold Cross Validation

We have performed a five-fold cross-validation on our dataset. Five-fold stratified cross-validation yielded a mean accuracy of 94.39% ± 1.09%, a macro F1-score of 94.38% ± 1.09%, and an AUC of 0.9969 ± 0.0015, demonstrating strong generalization and stability across data partitions. The near-perfect AUC indicates robust class discrimination, with minor variance in accuracy attributable to decision boundary sensitivity rather than model instability.

### 4.8. Explainability Analysis via GRAD-CAM

**Figure 5:**
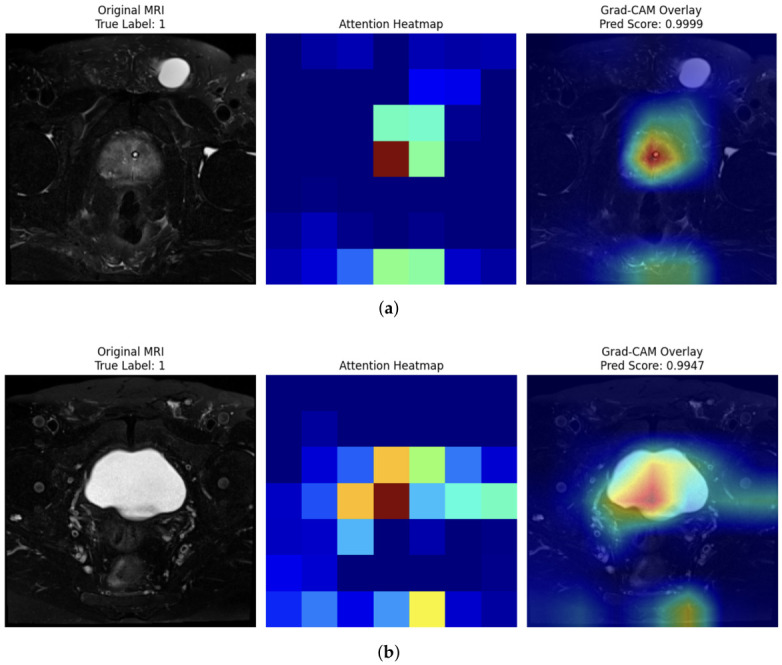
Grad-CAM explainability analysis on T2-weighted MRI test samples. (**a**) The model correctly classifies a clinically significant lesion (True Label: 1) with a near-perfect prediction score of 0.9999. (**b**) Another positive case (True Label: 1) is correctly identified with a prediction score of 0.9947.

Figure 5 presents representative Grad-CAM results on T2-weighted MRI test samples. Each subfigure consists of three panels: the original MRI slice with its ground-truth PI-RADS label, the raw attention heatmap generated from the target layer, and the Grad-CAM overlay superimposed on the original image. In Figure 5a, the model correctly classifies a clinically significant lesion (True Label: 1) with a near-perfect prediction score of 0.9999. The attention heatmap reveals a tightly localized activation centered within the prostate gland, and the Grad-CAM overlay confirms that the model’s focus is precisely concentrated on the suspicious lesion region, with the highest-intensity activation (red) corresponding to the lesion core and progressively diminishing activation (yellow–green–blue) toward the peripheral zones. In Figure 5b, another positive case (True Label: 1) is correctly identified with a prediction score of 0.9947. Here, the heatmap exhibits a broader activation pattern consistent with the larger hyperintense prostate region visible on the T2 slice, with the model appropriately directing maximum attention (dark red) toward the central gland and transition zone—anatomical regions commonly associated with clinically significant prostate cancer on T2-weighted imaging.

### 4.9. Comparative Study with the State-of-the-Art Models

An in-depth comparative analysis of the proposed DSPCD framework was performed in comparison with a number of state-of-the-art (SOTA) models dedicated to the classification of prostate cancer. The results of this benchmark are summarized in Table 5. Comparison is taken between a diverse range of models, in-depth learning architectures such as DTL-PSCC [38], and the Texture Graph Transformer [39], and traditional machine learning methods such as SIFT [40], Random Forest [41], and Tabnet [42].

### 4.10. Comparative Performance on the PI-CAI Dataset

As demonstrated in Table 6, the proposed DSPCD model significantly outperforms existing benchmarks. While the compared methods rely on bi-parametric (bpMRI) or multi-parametric (mpMRI) protocols—which include functional sequences like ADC and DWI—our framework achieves superior case-level AUC (0.96 and 0.98) using exclusively T2W sequences. This indicates that the dual-stream architecture successfully extracts high-level discriminative features from anatomical data that are typically only captured through multiple modalities in other frameworks.

## 5. Conclusions

This study proposed a dual-stream attention network to classify PCa from T2-weighted MRI, achieving high diagnostic accuracy on a sample of 3850 images of Saudi Arabian patients. Our model had an accuracy of 97.8% on the validation data and 96.4% on the test data with an AUC of 0.99. The breakthrough innovation of our architecture, the Channel-Spatial Attention and Adaptive Feature Fusion modules, allowed the extraction and fusion of salient multi-scale features effectively, which is required to detect small pathological changes in prostate MRI data.

### Limitations and Future Directions

We acknowledge certain limitations, such as the retrospective nature and inclusion of data in only one geographic area (Jazan), which limit the extrapolation of our results to the rest of the Saudi population. Second, the model was explicitly trained on T2-weighted images only, unlike the multi-parametric MRI (mpMRI), which includes DWI and DCE sequences, as the clinical gold standard for PCa diagnosis. Despite the good performance in T2-weighted images, it is possible that the incorporation of multi-parametric data can benefit the performance of the model.

Future work focuses on validating the model through prospective, multi-center trials across Saudi hospitals and extending it to incorporate full multiparametric MRI sequences for improved PI-RADS alignment. Future work will also focus on head-to-head comparisons between the DSPCD model and leading commercial prostate MRI analysis tools. Benchmarking against these industry standards is a vital step toward validating our model’s clinical readiness and diagnostic superiority.

Overall, the suggested dual-stream attention network provides a powerful and precise tool to classify PCa in an automated system in a Saudi Arabian context. This input will contribute to a DL-based architecture as well as provide a clinical tool that has the possibility to dramatically change the diagnostic course of PCa in the Kingdom and elsewhere.

## Figures and Tables

**Table 1:** Dataset Hierarchical Summary: Series and Slice Distribution (2022–2025).

PI-RADS	Patients	Total Series	Total Slices	Avg. Series/Pat	Avg. Slices/Ser
2–3	36	560	61,720	15.56	110.21
4–5	39	623	68,623	15.97	110.15
**Total**	**75**	**1183**	**130,343**	**15.77**	**110.18**

**Table 2:** Distribution of T2-weighted Image Series by Acquisition Protocol.

Series Description	Number of Images
t2_FS-TRA (Fat Suppressed Transverse)	1842
t2_tse_TRA (Turbo Spin Echo Transverse)	1762
t2_TRA (Standard Transverse)	101
t2_haste_tra_ (Half-Fourier Single-shot Turbo)	45
t2_tse_TRA-rectum (Turbo Spin Echo with Rectum)	35
t2_haste_TRA (Half-Fourier Single-shot Turbo)	35
t2_FS_TRA (Fat Suppressed Transverse)	30
**Total T2-weighted Images**	**3850**

**Table 3:** Complete Dual-Stream Attention Network Architecture and Layer Specifications.

Layer (Type)	Output Shape	Parameters	Connected To
input_layer	(None, 224, 224, 3)	0	-
data_augmentation (Sequential)	(None, 224, 224, 3)	0	input_layer
*Data Augmentation Pipeline*			
Horizontal Flip	(None, 224, 224, 3)	0	data_augmentation
Rotation (±15°)	(None, 224, 224, 3)	0	data_augmentation
Zoom (±10%)	(None, 224, 224, 3)	0	data_augmentation
stack (Recombine)	(None, 224, 224, 3)	0	augmentation outputs
add (ImageNet Preprocessing)	(None, 224, 224, 3)	0	stack
*Backbone Feature Extraction*			
resnet50 (Functional)	(None, 7, 7, 2048)	23,587,712	preprocessing
*Stream 1: Local Features (3 × 3 Kernel)*			
conv2d_440 (Conv2D 3 × 3, 256 filters)	(None, 7, 7, 256)	4,718,848	resnet50
channel_spatial_attention_1	(None, 7, 7, 256)	16,771	conv2d_440
(ChannelSpatialAttention)			
*Stream 2: Global Features (5 × 5 + Depthwise)*			
conv2d_442 (Conv2D 5 × 5, 256 filters)	(None, 7, 7, 256)	13,107,456	resnet50
depthwise_conv2d_8 (DepthwiseConv2D 3 × 3)	(None, 7, 7, 256)	2560	conv2d_442
channel_spatial_attention_2	(None, 7, 7, 256)	16,771	depthwise_conv2d_8
(ChannelSpatialAttention)			
*Cross-Stream Fusion*			
multiply_7 (Element-wise Multiply)	(None, 7, 7, 256)	0	CSA_1, CSA_2
add_20 (Multi-path Aggregation)	(None, 7, 7, 256)	0	CSA_1, CSA_2, multiply
batch_normalization (BatchNorm)	(None, 7, 7, 256)	1024	add_20
*Adaptive Feature Fusion*			
adaptive_feature_fusion	(None, 7, 7, 256)	131,584	batch_normalization
(AdaptiveFeatureFusion)			
*Global Pooling*			
global_average_pooling2d	(None, 256)	0	adaptive_feature_fusion
global_max_pooling2d	(None, 256)	0	adaptive_feature_fusion
concatenate_19 (Concatenate)	(None, 512)	0	GAP, GMP
*Classification Head*			
dense_94 (Dense 512 units, ReLU)	(None, 512)	262,656	concatenate
dropout_40 (Dropout rate 0.4)	(None, 512)	0	dense_94
dense_95 (Dense 256 units, ReLU)	(None, 256)	131,328	dropout_40
dropout_41 (Dropout rate 0.3)	(None, 256)	0	dense_95
dense_96 (Dense 1 unit, Sigmoid)	(None, 1)	257	dropout_41

**Table 4:** Per-class classification metrics for test and validation datasets. Precision represents the ratio of positive predictions that are true, recall represents the ratio of the true positive cases that are correctly identified, and the F1-score is the harmonic mean of precision and recall.

Class	Dataset	Precision	Recall	F1-Score	Support
PIRADS 2–3	Test	0.96	0.96	0.96	275
PIRADS 2–3	Validation	0.99	0.98	0.98	287
PIRADS 4–5	Test	0.96	0.96	0.96	311
PIRADS 4–5	Validation	0.98	0.99	0.98	289
Macro Average	Test	0.96	0.96	0.96	586
Macro Average	Validation	0.98	0.98	0.98	576
Weighted Average	Test	0.96	0.96	0.96	586
Weighted Average	Validation	0.98	0.98	0.98	576

**Table 5:** A comparative analysis of a proposed DSDLP model with prevailing state-of-the-art techniques for prostate cancer classification.

Reference	Model	Accuracy
[38]	DTL-PSCC	85.09
[39]	Texture graph transformer	89.5
[40]	SIFT	95
[41]	Random Forest	85
[42]	Tabnet	89.9
Proposed	DSPCD (Validation)	97.8
Proposed	DSPCD (Test)	96.4

**Table 6:** Comparative performance analysis on the PI-CAI and Prostate158 datasets using case-level AUC metrics.

Reference	Model Architecture	Protocol	Dataset	AUC
[43]	Z-SSMNet (Zonal-aware)	bpMRI	PI-CAI	0.90
[44]	Spatial Transformer Alignment	bpMRI	PI-CAI	0.93
[45]	Dual-branch Slice-based	bpMRI	PI-CAI	0.90
[46]	Harmonized (T2W + ADC + HBV)	bpMRI+	PI-CAI	0.85
[47]	SOGA (Geometric Attention)	bpMRI	Prostate158	0.75
[48]	Weakly Supervised	mpMRI/bpMRI	Prostate158	0.78
[49]	Swin Transformer (3-channel)	mpMRI	Prostate158	0.85
**Proposed**	**DSPCD**	**T2W Only**	**PI-CAI**	**0.96**
**Proposed**	**DSPCD**	**T2W Only**	**Prostate158**	**0.98**

## Data Availability

The data supporting the conclusions of this article will be made available by the authors on request.
